# C677T polymorphism in the *methylenetetrahydrofolate reductase* gene is associated with primary closed angle glaucoma

**Published:** 2008-03-26

**Authors:** Shazia Michael, Raheel Qamar, Farah Akhtar, Wajid Ali Khan, Asifa Ahmed

**Affiliations:** 1 Department of Biosciences, Comsats Institute of Information Technology, Islamabad, Pakistan; 2 Al-Shifa Trust Eye Hospital, Jhelum Road, Rawalpindi, Pakistan

## Abstract

**Purpose:**

To determine whether or not there is an association of the *methylenetetrahydrofolate reductase* (*MTHFR*) C677T polymorphism with disease in cohorts of primary open-angle glaucoma (POAG) and primary closed-angle glaucoma (PCAG) from Pakistan.

**Methods:**

This was a prospective study consisting of 150 patients (90 POAG and 60 PCAG) and 70 control subjects. Genomic DNA was extracted from leukocytes of the peripheral blood. *MTHFR* C677T polymorphism analysis was performed by the polymerase chain reaction-restriction fragment length polymorphism (RFLP) technique.

**Results:**

The prevalence of the *MTHFR* C/T genotype was 22.2% in POAG, 13.3% in PACG, and 18.6% in controls whereas the *MTHFR* T/T genotype was present solely in the PACG group (6.9%). The difference regarding the T/T genotype between PACG and controls was statistically significant (p<0.01).

**Conclusions:**

The *MTHFR* C677T polymorphism was found to be associated with PCAG but not POAG in patients of Pakistani origin.

## Introduction

Glaucoma is the second leading cause of blindness worldwide, affecting over 70 million individuals. The most common types of glaucoma include primary open-angle glaucoma (POAG) and primary closed-angle glaucoma (PCAG) [1,2].

Pathophysiologically, POAG is a progressive optic nerve disease often associated with elevated intraocular pressure (IOP) and characterized by optic disc cupping and visual field loss [3]. In PCAG, anatomic features act in concert to cause swallowing of the anterior chamber. As a patient ages, the thickening of the crystalline lens leads to a relative pupil block that puts the iris into apposition with the trabecular meshwork or cornea. Chronic angle closure denotes an angle with areas that are closed permanently with peripheral anterior synechia [4].

An increased level of plasma homocysteine has been observed in patients with glaucoma [5]. Homocysteine can induce vascular injuries [6], alterations in the extracellular matrix [7], and neuronal cell death by inducing apoptosis or excitotoxicity [8,9]. More recently, hyperhomocysteinemia has been shown to be involved in the structural remodeling of connective tissues [10]. Previous evidence has suggested restructuring of the sclera in acute PCAG [11].

The risk of PCAG has been reported to be increased in Eskimos as well as Chinese and Asian Indians [12]. The studies predispose a genetic link to the development of PCAG in these populations. Homocysteine concentrations have been found to be affected by a single base pair mutation, C677T, in the *methylenetetrahydrofolate reductase* (*MTHFR*) gene [13,14]. MTHFR catalyzes the reduction of 5,10-methylenetetrahydrofolate to 5-methylenetetrahydrofolate, the major circulatory form of folate and methyl donor for homocysteine remethylation. Bleich and coworkers found a raised plasma homocysteine level and C677T polymorphism in Caucasian glaucoma patients. This was the first study to provide evidence of association of *MTHFR* C677T polymorphism with Hcy level and open-angle glaucoma [15]. Junemann et al. [16] also reported increased frequency of the C677T polymorphism of *MTHFR* in patients with open-angle glaucoma but not in patients with pseudoexfoliation syndrome (PEXG). No association of *MTHFR* C677T polymorphism with normal tension glaucoma (NTG) and POAG have been observed in the Japanese population [17] and in a Central European population [18]. However, more recently, C677T polymorphism of *MTHFR* has been found to be a genetic risk factor of NTG in the Korean population [19]. The current data suggests that the prevalence of this polymorphism may vary in different ethnic populations and also among the different types of glaucoma. To date, no studies with regard to the association of *MTHFR* genotype with PCAG have been investigated.

In this study, we aimed to identify whether there is an association of the *MTHFR* C677T polymorphism with POAG and PCAG in the Pakistani population. We were able to show a significant association of the C677T polymorphism with PCAG but not with POAG.

## Methods

### Patient selection criteria

In our prospective study, we assessed a total of 180 Pakistani subjects comprised of 90 patients with POAG, 60 patients with PCAG, and 70 control subjects. All patients were recruited from the Christian Hospital, Taxila, Pakistan following the approval from the Hospital Ethical Committee. Only patients who fulfilled the selection criteria and gave written informed consent in line with the Declaration of Helsinki were included in the study. All patients underwent a complete ophthalmic examination, which included slit lamp biomicroscopy, testing for best corrected visual acuity with the help of Snellen’s chart, visual field defects determined with Humphrey 30–2, Goldman applanation tonometer to measure intraocular pressure (IOP), indirect fundoscopy to determine cup-to-disc ratio (c/d), and to assess type of glaucoma.

POAG was defined by the following criteria: IOP of more than 21 mmHg, typical glaucomatous cupping of the optic disc (diffuse or focal thinning of the disc rims and cup-to-disc ratio of more than 0.5), visual field defect typical of glaucoma, an open anterior chamber angle, and no family history of glaucoma. PCAG was defined by the same criteria as POAG except that in PCAG, a gonioscopically closed anterior chamber angle was noted. Patients were excluded if they had any other ocular diseases or a personal history of hypertension, diabetes, or cardiovascular disease.

Control subjects were healthy Pakistani individuals above the age of 35 years and had no history of any eye problems, hypertension, diabetes, or cardiovascular diseases. All controls underwent a gonioscopic examination, had an IOP of less than 21mmHg, and a c/d ratio of less than 0.5.

### Polymerase chain reaction-restriction fragment length polymorphism

Peripheral blood was collected in acid citrate dextrose (ACD) tubes to prevent coagulation of blood samples. Genomic DNA was extracted from whole blood using the standard phenol-chloroform method. Detection of C677T *MTHFR* polymorphism was performed by polymerase chain reaction (PCR) followed by HinfI restriction enzyme digestion. Briefly, we used the forward primer 5′-CCT TGA ACA GGT GGA GGC CAG-3′ and the reverse primer 5′-GCG GTG AGA GTG GGG TGG AG-3′ to amplify a 294 base pair (bp) fragment of the *MTHFR* gene. Each 25 μl PCR reaction contained 2.5 μl of 10X reaction buffer, 1.5 mmol MgCl_2_ (Fermentas, Glen Burnie, MD), 2 μl from 10 pmol of each primer, 0.2 mmol of the deoxynucleoside triphosphates, 1 U of *Taq* DNA polymerase (Fermentas), and 100 ng of genomic DNA template. The mixture was denatured at 95 °C for 10 min, and the PCR reaction was performed for 35 cycles in a thermocycler (Eppendorf AG, Hamburg, Germany) under the following conditions: denaturation at 95 °C for 1 min, annealing at 65 °C for 30 s, and extension at 72 °C for 1 min. The final extension cycle of 72 °C was for 7 min. The PCR products were electrophoresed on an agarose gel (2%) to confirm the correct amplicon size. Restriction enzyme digestion was performed on PCR products using the HinfI restriction enzyme (Fermentas) following the suppliers protocol. After digestion, all fragments were resolved on an agarose gel (3%). A single fragment of 294 base pairs (bp) was identified as homozygous (CC); three fragments of 294, 168, and 126 bp were identified as heterozygous (CT); and two fragments of 168 and 126 bp were identified as homozygous (TT) genotype.

### Statistical analysis

Statistical analysis was performed using SPSS 12.0 for Windows (SPSS version 12.0, Chicago, IL). The odds ratio (OR) and 95% confidence interval (95% CI) were calculated by logistic regression. Allele frequency differences between POAG or PCAG patients and the controls were compared using Fisher’s exact test, and the genotype frequency differences between POAG or PCAG patients and the controls were compared by the Χ^2^ test. The criterion for statistical significance was p<0.05. Power analysis was performed with G*Power software version 3.0.8 [20].

## Results

To assess the association of C677T polymorphism of *MTHFR* in the Pakistani population, we took 90 POAG patients with the mean age of 57.9±14.3 years (64.4% males and 35.5% females), 60 PCAG patients with the mean age 57±10.05 years (51.67% males and 48.3% females), and 70 control subjects with the mean age of 50.9±8.1 years (66% males and 34% females).

The *MTHFR* genotype and allele frequencies were in Hardy–Weinberg equilibrium in both the patients of POAG and PCAG and the control subjects. The allele frequencies of the C677T variant in the POAG, PCAG, and control subjects are shown in Table 1. The genotype frequencies of the *MTHFR* C677T polymorphism in the POAG, PCAG, and control subjects are shown in Table 2. In POAG subjects, 20 samples (22.2%) were heterozygous having the CT genotype while 70 samples (77.7%) were homozygous with genotype CC. There was no sample with the TT genotype in the POAG subjects. In the PCAG subjects, four (6.7%) were homozygous with the TT genotype, 48 (80%) had the CC genotype, and eight (13.3%) had the CT genotype. In the control subjects, it was observed that 57 (81.4%) of the samples had no mutation and showed CC homozygosity whereas 13 (18.6%) showed the CT genotype. A significant difference in the *MTHFR* genotype frequencies between PCAG and the control subjects was observed (Χ^2^=12.96, p<0.01). However, no significant difference between the genotype frequencies of POAG and control subjects was found (Χ^2^=1.23, p=0.27).

**Table 1:** Allele frequencies of the C677T variation in *MTHFR* in control subjects and in POAG and PCAG patients.

***MTHFR* allele at nucleotide 677**	**Controls (n=70)**	**POAG (n=90)**	**p-Value**	**PCAG (n=60)**	**p-Value**
C	127 (90.7)	160 (84.1)	0.71	104 (86.7)	0.33
T	13 (9.2)	20 (15.9)		16 (13.3)	

POAG indicates primary open-angle glaucoma and PCAG indicates primary closed-angle glaucoma. Data are given as numbers (percentage). P-values were calculated using Fisher's exact test.

**Table 2:** Genotype frequencies of the C677T variation in *MTHFR* in control subjects and in POAG and PCAG patients.

***MTHFR* genotype at nucleotide 677**	**Controls (n=70)**	**POAG (n=90)**	**p-Value**	**PCAG (n=60)**	**p-Value**
TT	0 (0.0)	0 (0.00)	0.23	4 (6.7)	>0.01
CT	13 (18.57)	20 (22.2)		8 (13.3)	
CC	57 (81.4)	70 (77.8)		48 (80)	

POAG indicates primary open-angle glaucoma and PCAG indicates primary closed-angle glaucoma. Data are given as numbers (percentage). The p-values were calculated using the Χ^2^ test.

## Discussion

Our study is the first to report whether there is an association of *MTHFR* C677T polymorphism with PCAG. We have found a significant association of the *MTHFR* C677T polymorphism with PCAG. This finding suggests that the *MTHFR* polymorphism is a genetic risk factor for PCAG in patients of Pakistani origin.

Previous studies documented in literature have been performed on PEXG, NTG, and POAG. The association of *MTHFR* C677T with POAG, NTG, and PEXG still remains controversial. Ethnic differences appear to be the main governing factor behind the varying *MTHFR* genotype distribution and allele frequencies reported [16-19,21,22]. No association of the polymorphism has been found with PEXG [16,17]. We did not find any association of *MTHFR* polymorphism with POAG in the cohorts of Pakistani patients. Similar results have been obtained in a population of Iowa [21]. Recently, Zetterberg et al. also documented no significant differences between the control and the POAG group for the *MTHFR* 677T allele frequency or for the homozygous *MTHFR* 677TT genotype [22]. In the study conducted by Mabuchi et al. [17], genotype distribution of homozygosity (TT) and heterozygosity (CT) was 20.3% and 41.4%, respectively, for Japanese POAG patients. Similarly, Mossbock et al. [18] observed that the genotype distribution of TT was 6.9% and CT was 34.8%. We found the CT genotype distribution to be 22.2% but did not find the TT genotype in any of the 90 POAG samples. The difference in distribution could indeed be due to the small sample size. However, to date only Junemann et al. [16] have reported a positive association of *MTHFR* with POAG and they investigated a smaller sample size than ours with 76 patients and 71 controls.

The trabecular meshwork (TM) is a specialized tissue located at the anterior angle of the eye. Together with Schlemm’s canal (SC) and aqueous veins, it forms the major outflow/drainage pathway for the aqueous humor [23]. Widespread changes in the trabecular structures have been noted in chronic PACG. Sihota et al. [24] have recently reported an altered trabecular architecture evidenced by the presence of pigment granules within fused beams, the reduction of endothelial cells, and the pleomorphism of remaining endothelial cells. In addition, a progressive occurrence of fibrosis and degeneration in the trabecular meshwork with a compressed and obliterated Schlemm’s canal beneath iris adhesions has been observed [25]. We propose that these changes could be partly due to homocysteine-impaired metabolism. The TM is composed of connective tissues mainly collagen and elastic fibers. Homocysteine has been found to alter the connective tissue architecture and to instigate remodeling by contracting collagen gels [10]. Additionally, homocysteine causes alterations in extracellular matrix remodeling by inducing the expression of collagen and the α-actin level at the gene transcription level [11]. The homozygous mutation TT of *MTHFR*, which is observed in the PCAG subjects in this study, is reported to cause higher homocysteine levels than the CC or CT genotype [15]. The toxic effects of homocysteine have been specially observed on fibrillin 1 [26], a protein immunolocalized in the elastic fibers [27]. By remodeling the connective tissue, homocysteine levels may be involved in enhancing the attachment of the TM to the iris, a phenomenon typical of chronic PCAG. In addition to the TM, fibrillin has been immunolocalized to the connective tissues of the anterior segment including the conjunctival, iris and ciliary body stroma, the ciliary processes, the corneal stroma and corneal epithelial basement membrane, and the endothelium of Schlemm’s canal [28]. Therefore, the effect of hyperhomocysteine on fibrillin 1 could affect the overall anterior segment structure during development and aging as seen in PCAG. A recent study by Jong-Wang et al. has suggested a remodeling of the sclera in acute PCAG [11].

We conclude that in the development of chronic PCAG, the *MTHFR* polymorphism may be playing an essential role in remodeling the TM and anterior segment connective tissue. This possibility warrants further study.
